# The Global and Regional Prevalence of Hospital-Acquired Carbapenem-Resistant *Klebsiella pneumoniae* Infection: A Systematic Review and Meta-analysis

**DOI:** 10.1093/ofid/ofad649

**Published:** 2023-12-19

**Authors:** Xing-chen Lin, Chang-li Li, Shao-yang Zhang, Xiao-feng Yang, Meng Jiang

**Affiliations:** Emergency and Trauma Center, The First Affiliated Hospital, Zhejiang University School of Medicine, Hangzhou, China; Department of FSTC Clinic, The First Affiliated Hospital, Zhejiang University School of Medicine, Hangzhou, China; Emergency and Trauma Center, The First Affiliated Hospital, Zhejiang University School of Medicine, Hangzhou, China; Emergency and Trauma Center, The First Affiliated Hospital, Zhejiang University School of Medicine, Hangzhou, China; Emergency and Trauma Center, The First Affiliated Hospital, Zhejiang University School of Medicine, Hangzhou, China

**Keywords:** carbapenem-resistant *Klebsiella pneumoniae*, hospital-acquired infections, prevalence, risk factor, systematic review

## Abstract

**Background:**

Due to scarce therapeutic options, hospital-acquired infections caused by *Klebsiella pneumoniae* (KP), particularly carbapenem-resistant KP (CRKP), pose enormous threat to patients’ health worldwide. This study aimed to characterize the epidemiology and risk factors of CRKP among nosocomial KP infections.

**Method:**

MEDLINE, Embase, PubMed, and Google Scholar were searched for studies reporting CRKP prevalence from inception to 30 March 2023. Data from eligible publications were extracted and subjected to meta-analysis to obtain global, regional, and country-specific estimates. To determine the cause of heterogeneity among the selected studies, prespecified subgroup analyses and meta-regression were also performed. Odds ratios of CRKP-associated risk factors were pooled by a DerSimonian and Laird random-effects method.

**Results:**

We retained 61 articles across 14 countries and territories. The global prevalence of CRKP among patients with KP infections was 28.69% (95% CI, 26.53%–30.86%). South Asia had the highest CRKP prevalence at 66.04% (95% CI, 54.22%–77.85%), while high-income North America had the lowest prevalence at 14.29% (95% CI, 6.50%–22.0%). In the country/territory level, Greece had the highest prevalence at 70.61% (95% CI, 56.77%–84.45%), followed by India at 67.62% (95% CI, 53.74%–81.79%) and Taiwan at 67.54% (95% CI, 58.65%–76.14%). Hospital-acquired CRKP infections were associated with the following factors: hematologic malignancies, corticosteroid therapies, intensive care unit stays, mechanical ventilations, central venous catheter implantations, previous hospitalization, and antibiotic-related exposures (antifungals, carbapenems, quinolones, and cephalosporins).

**Conclusions:**

Study findings highlight the importance of routine surveillance to control carbapenem resistance and suggest that patients with nosocomial KP infection have a very high prevalence of CRKP.

One of the most common gram-negative opportunistic pathogens, *Klebsiella pneumoniae* (KP) usually causes pneumonia, blood infections, and other nosocomial infections (eg, hepatic abscess and urinary tract infection) [1]. Antimicrobial resistance has become a global catastrophe posing threats to public health. The excessive and inappropriate use of broad-spectrum antibiotics in recent years has dramatically increased KP's drug resistance [2]. After extended-spectrum β-lactamase–producing KP strains, carbapenem-resistant KP (CRKP) isolates emerged as a major global public health concern that is associated with high morbidity and mortality rates [2, 3]. According to the World Health Organization, developing new and effective antibiotic medicines against CRKP is necessary [3].

In previous meta-analyses, the pooled mortality rate related to CRKP infections was estimated to range from 37.2% (95% CI, 33.1%–41.4%) to 42.1% (95% CI, 37.1%–47.3%) [4, 5]. In Europe, CRKP accounted for >90 000 infections and >7000 deaths annually [6]. For other regions, including Malawi, up to 75% of KP blood infections are now multidrug resistant [7]. To estimate the epidemiology of nosocomial CRKP infections, several studies have been conducted. The prevalence of CRKP causing nosocomial infections varies remarkably among different studies depending on the study's variables (eg, region and period, cohorts, sample size, and clinical wards). Despite limited representative reports, CRKP prevalence has been rapidly increasing in China from 3% in 2005 to 21% in 2017 [8]. Moreover, in European countries, antibiotic resistance surveillance revealed that CRKP infection percentage increased significantly from 6.2% in 2012 to 8.1% in 2015 [9]. From a meta-analysis of 39 retrospective studies, carbapenem resistance in KP has a pooled mortality rate of 24.0% in Iran [10].

Despite these reports, the true global epidemiology of CRKP remains unclear. A comprehensive understanding of the current epidemiology of hospital-acquired CRKP infection is therefore warranted. To better understand the current burden and trend worldwide, this systematic review aimed to comprehensively analyze the data available on the prevalence of hospital-acquired infections with CRKP. This will provide an updated global estimate and information on the development of context-specific control interventions against this dangerous pathogen.

## METHODS

This systematic review and meta-analysis was conducted according to the PRISMA guidelines (Preferred Reporting Items for Systematic Reviews and Meta-analyses) and the GATHER statement (Guidelines for Accurate and Transparent Health Estimates Reporting) [11, 12].

### Search Strategy and Selection Criteria

To determine studies on CRKP prevalence in hospital-acquired KP infections from the database inception until 30 March 2023, MEDLINE, Embase, PubMed, and Google Scholar were systematically researched. Search terms included combinations of keywords and controlled vocabulary terms related to hospital-acquired infections and KP (Supplementary Table 1). No language or geographic restrictions were applied. Reference lists of relevant (systematic) reviews [5, 10, 13, 14] were also screened for eligible studies.

### Inclusion and Exclusion Criteria

This study comprised epidemiologic studies that provide data on the prevalence of hospital-acquired CRKP infections, regardless of whether the studies were prospectively or retrospectively designed. According to the standard of the National Healthcare Safety Network (Centers for Disease Control and Prevention), hospital-acquired CRKP infection was defined as infection that occurred 48 hours after admission [15]. Studies were included that reported relevant data from patient cohorts in entire departments/wards or hospitals. Studies were excluded if they (1) were conducted in specific diseases or patient subgroups; (2) were reviews/systematic reviews, letters, experimental studies, editorials, case reports, case-control studies, and randomized controlled trials; and (3) made no clear distinction between CRKP infection and colonization status. Studies comprising non–disease-specific cases from whole hospitals or individual wards were included, but those with specific cases were excluded (eg, transplant, malignancy). This ensured the representativeness of our estimates for these institutions and wards.

Two reviewers (M. J. and C.-L. L.) independently screened all identified titles and abstracts. Full texts of potentially relevant studies were downloaded or requested from corresponding authors and further reviewed.

### Data Extraction and Outcomes of Interest

Using a predesigned form, 2 authors (C.-L. L. and X.-C. L.) extracted relevant data. All data extraction discrepancies were resolved by consensus of those who did the initial review (M. J. and C.-L. L.). The primary outcome was CRKP prevalence among all patients with hospital-acquired KP infection. A case of KP infection was categorized as “carbapenem resistant” when the authors stated so or when carbapenem resistance data of KP isolates were reported in the studies. The strain was categorized as “carbapenem resistant” when antimicrobial susceptibility testing showed resistance to at least 1 type of carbapenem (eg, ertapenem, meropenem, and imipenem) by either disc diffusion or broth microdilution methods.

According to the region set of the Global Burden of Disease (GBD) study, countries were categorized into distinct regions [16]. Based on the 2019 sociodemographic index (SDI)—a summary indication of overall development based on total fertility rate, average years of schooling, and income per capita—countries were classified into high SDI (≥0.805) and low and middle SDI (<0.805) [17]. Based on multivariable logistic regression, a subset of publications explored the associated risk factors for CRKP infection. Thus, the corresponding odds ratios of the risk factors were extracted from these articles.

### Statistical Analysis

#### Estimation of the Global Prevalence of CRKP

One of the authors (M. J.) extracted the summary estimates from the eligible studies, and another author (S.-Y. Z.) independently verified these data. Additionally, discrepancies were resolved by discussion. The extracted information included first author name, publication year, study's country/region and period, study design, description of the medical institution/wards, culture-positive sample's source, methods of antimicrobial susceptibility tests, total sample of hospital-acquired KP infection cases, number of CRKP cases, and definition of hospital-acquired infection. The corresponding authors of the studies were contacted by email for clarifications or missing data.

The quality of studies was assessed by a quality scale (Supplementary Table 2) developed per the STROBE statement (Strengthening the Reporting of Observational Studies in Epidemiology) [18, 19]. The quality scale consisted of 4 items: sample population, cohort size, outcomes assessment, and analytic methods. Each part can be assigned 0 (low quality), 1 (moderate), or 2 (high). Each study could be scored up to 8 points. Publications with a total score of 6 to 8 are deemed high quality, whereas those with a total score of 3 to 5 and 0 to 2 are deemed moderate and low quality, respectively.

To estimate the pooled prevalence of hospital-acquired CRKP infection among patients with KP infection, a meta-analysis of the reports was performed, with variance estimates generated by the Freeman-Tukey double-arcsine transformation that has been validated in previous studies [20, 21]. A random- or fixed-effects model was used according to the heterogeneity among the selected studies. Heterogeneity among studies was calculated by Cochran *Q* (represented as χ^2^ and *P* values) and the *I*^2^ statistic, which represents the percentage of variation among studies that causes heterogeneity. Because heterogeneity was high in this study (*I*^2^ > 50%, *P* < .01), random-effects models were used for summary statistics. To determine the geographic differences in CRKP prevalence, a map of pooled prevalence was generated by Stata version 17.0 (StataCorp). If the research took several years, the last year was used as the reference for estimating point prevalence. We evaluated the publication bias by using the funnel plot and Egger test. Sensitivity analysis was performed by excluding any single study to test the robustness of our findings.

Potential sources of heterogeneity were explored further by subgroup and meta-regression analyses. To investigate which factors caused heterogeneity in this study, related factors in single- and multiple-variable models were examined. The investigated factors were study period, geographic region, SDI level, methods of antimicrobial susceptibility tests, patient cohort, study design, sample source, and sample size.

#### Meta-analysis of Associated Risk Factors of Hospital-Acquired CRKP Infection

Based on the DerSimonian and Laird random-effects method of meta-analysis, the odds ratios of associated risk factors for CRKP infection, as reported in at least 2 individual reports, were synthesized [22].

All analyses were conducted with Stata version 17.0 and R studio software (version 4.2.2). *P* < .05 was considered statistically significant.

## RESULTS

**Figure 1.**
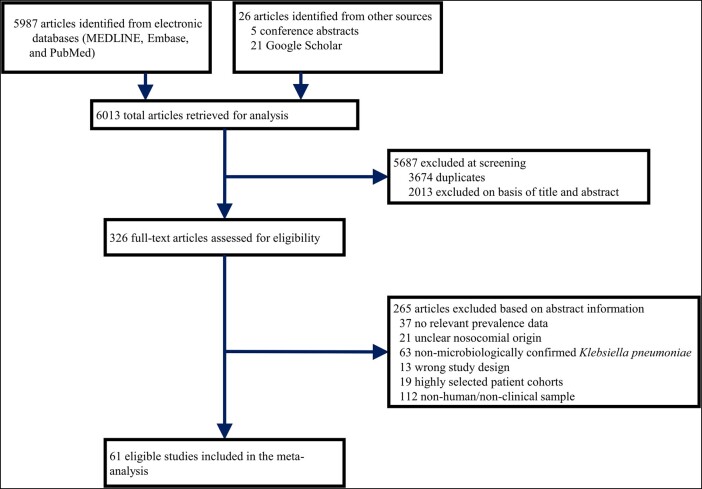
Flow diagram of study selection.

The literature search identified 6013 records, out of which 61 articles were eligible for prevalence calculations (Figure 1; Supplementary Table 3) [23–83]. Characteristics of the studies are provided in Table 1. The quality assessment for each study is provided in Supplementary Table 4. The 61 studies in CRKP prevalence analysis comprised a sample population of 513 307 individuals (median, 272 [IQR, 104–706]; range, 23–277 758) from 61 study populations in 14 countries or territory, which were affiliated to 5 GBD regions: East Asia (24 study populations), high-income North America (6), North Africa and Middle East (14), South Asia (4), and Western Europe (13). Study years ranged from 2003 to 2020 and publication years ranged from 2008 to 2023. On average, articles were published 5.4 years after the year that the study was started. Most reports were from mainland China (23 studies), followed by Iran (11 studies). According to our quality criteria, 38 publications were high quality (6–8 points), 22 were moderate quality (3–5 points), and the remaining 1 publication was low quality (2 points) (Supplementary Table 4).

**Table 1.** Characteristics of the Studies

	Study					HAI, No. of Cases				
First Author	Period	Location	Study Design	Medical Institution	Culture-Positive Sample Source	KP	CRKP	HAI Definition	Antimicrobial Susceptibility Tests Methods	Quality Score
Huang [23]	2020–2021	China (Hubei)	Prevalence, single center	Teaching hospital with 5200 beds	Various	109	24	Infection occurring 48 h after admission	Disc diffusion	4
Liu [24]	2019–2021	China (Beijing)	Prevalence, single center	Teaching hospital with 1000 beds	Various	30	19	CDC/NHSN criteria	Broth microdilution	4
Huang [25]	2014–2020	China (Zhejiang)	Prevalence, single center	Teaching hospital with 4100 beds	Cerebrospinal fluid	36	16	Infection occurring 48 h after admission	Broth microdilution	5
Chen [26]	2012–2019	China (Hunan)	Prevalence, single center	Teaching hospital with 3500 beds	Blood	706	212	CDC/NHSN criteria	Broth microdilution	8
Wang [27]	2019–2020	China (Zhejiang)	Prevalence, single center	Teaching hospital with 1200 beds	Various	1573	417	Infection occurring 48 h after admission	Broth microdilution	7
Liu [28]	2018–2020	China (Hubei)	Prevalence, single center	Teaching hospital with 5200 beds	Blood	510	77	CDC/NHSN standard	Broth microdilution	8
Lin [29]	2006–2020	China (Zhejiang)	Prevalence, single center	Teaching hospital with 1100 beds	Various	3054	288	NA	Disc diffusion	6
Wang [30]	2017–2019	China (Hebei))	Prevalence, multicenter	43 tertiary hospitals in Hebei province	Various	44 382	6328	NA	Broth microdilution	6
Indrajith [31]	2017–2018	India	Prevalence, single center	Teaching hospital	Various	89	52	NA	Broth microdilution	4
Shankar [32]	2013–2018	India	Prevalence, multicenter	Seven centers in India	Various	290	230	NA	Disc diffusion	6
Shao [33]	2019	China (Shandong)	Prevalence, single center	Teaching hospital with 3889 beds	Lower respiratory tract secretions	258	31	Infection occurring 48 h after admission	Disc diffusion	7
Thapa [34]	2018–2019	Nepal	Prevalence, single center	NA	Various	58	35	NA	Disc diffusion	4
Chen [35]	2015–2020	China (Liaoning)	Prevalence, single center	A 2249-bed tertiary hospital	Various	491	51	NA	Disc diffusion	6
Zhou [36]	2019–2020	China (Shanghai)	Prevalence, single center	Teaching hospital with 800 beds	Various	1081	341	NA	Disc diffusion	6
Khairy [37]	2017	Egypt	Prevalence, multicenter	Two tertiary care hospitals with 800 and 120 beds	Various	42	24	CDC/NHSN standard	Broth microdilution	6
Chang [38]	2014–2018	China (Jiangsu)	Prevalence, single center	Teaching hospital with 3800 beds	Blood	285	46	Infection occurring 48 h after admission	Broth microdilution	7
Xiao [39]	2013–2015	China (Zhejiang)	Prevalence, single center	Teaching hospital with 5000 beds	Blood	371	104	CDC/NHSN criteria	Broth microdilution	6
Zhang [40]	2013–2019	China (Beijing)	Prevalence, single center	Teaching hospital with 2000 beds	Blood	496	108	Culture-proven infections	Broth microdilution	7
Hu [41]	2008–2018	China (Zhejiang)	Prevalence, multicenter	Hospitals are distributed across all 11 cities in Zhejiang province	Various	277 758	29 588	NA	Broth microdilution	6
Zhao [42]	2003–2016	China (Zhejiang)	Prevalence, single center	Teaching hospital with 3263 beds	Various	2299	214	NA	Broth microdilution	6
Kiaei [43]	2015–2017	Iran	Prevalence, multicenter	4 referral hospitals	Various	175	37	Infection occurring 48 h after admission	Broth microdilution	6
Li [44]	2014–2018	China (Henan)	Prevalence, multicenter	6 teaching hospitals with a combined 182 ICU hospital beds	Various	507	244	Infection occurring 48 h after admission	Broth microdilution	6
Balkhair [45]	2007–2016	Oman	Prevalence, single center	Teaching hospital with 600 beds	Blood	227	57	Infection occurring 48 h after admission	Broth microdilution	7
Messaoudi [46]	2012–2014	Tunisia	Prevalence, single center	NA	Various	2160	342	NA	Disc diffusion	6
Bahramian [47]	2014–2017	Iran	Prevalence, single center	NA	Urine	120	12	NA	Disc diffusion	3
Zare [48]	2015–2016	Iran	Prevalence, multicenter	Teaching hospitals of 2 cities	Various	23	5	NA	Disc diffusion	4
Mohammad [49]	2012	Iran	Prevalence, single center	A university hospital in Tehran	Various	53	5	Culture-proven infections	Disc diffusion	3
Dong [50]	2011–2014	China (Beijing)	Prevalence, single center	A 970-bed tertiary pediatric hospital	Blood	164	52	Culture-proven infections	Broth microdilution	6
Zheng [51]	2014–2016	China (Liaoning)	Prevalence, single center	Teaching hospital with 3700 beds	Blood	289	59	Culture-proven infections	Broth microdilution	7
Chen [52]	2014–2015	Taiwan	Prevalence, single center	NA	Various	114	77	NA	Broth microdilution	5
Zhang [53]	2011–2014	China (Beijing)	Prevalence, single center	A 970-bed tertiary pediatric hospital	Blood	138	54	Culture-proven infections	Broth microdilution	6
Koppe [54]	2011–2016	Germany	Prevalence, multicenter	655 hospitals	Various	154 734	975	NA	Broth microdilution	6
Moghadampour [55]	2017	Iran	Prevalence, single center	Alzahra hospital	Various	80	51	NA	Disc diffusion	4
Firoozeh [56]	2013–2014	Iran	Prevalence, single center	Shahid Beheshti Hospital	Various	181	48	NA	Disc diffusion	5
Han [57]	2014–2015	USA	Prevalence, multicenter	Kindred Healthcare network of LTACHs	Various	3470	821	CDC/NHSN criteria	Broth microdilution	8
Zhan [58]	2013–2015	China (Zhejiang)	Prevalence, single center	Teaching hospital with 3400 beds	Various	1838	140	NA	Broth microdilution	6
Veeraraghavan [59]	2015–2016	India	Prevalence, single center	Teaching hospital	Blood	115	73	Culture-proven infections	Disc diffusion	6
Ou [60]	2013–2015	China (Hubei)	Prevalence, single center	A 4854-bed tertiary pediatric hospital	Various	955	117	NA	Disc diffusion	6
Monari [61]	2014–2015	Italy	Prevalence, single center	Perugia General Hospital	Various	131	42	ECDC 2013	Broth microdilution	7
Xu [62]	2013	China (Beijing)	Prevalence, multicenter	208 hospitals	Blood	6061	333	NA	Broth microdilution	6
Trecarichi [63]	2010–2014	Italy	Prevalence, multicenter	13 Italian hematologic units	Blood	278	161	Infection occurring 48 h after admission	Disc diffusion	5
Conte [64]	2011–2013	Italy	Prevalence, multicenter	17 laboratories from 14 cities	Blood	461	167	NA	Broth microdilution	4
Eftekhar [65]	2011–2012	Iran	Prevalence, multicenter	Shariati Hospital and Motahari Burn Hospital	Various	55	5	NA	Disc diffusion	4
Fazeli [66]	2012–2013	Iran	Prevalence, single center	Main university hospital of Isfahan	Various	112	49	NA	Disc diffusion	5
Vardakas [67]	2006–2009	Greece	Prevalence, single center	350-bed tertiary center	Various	104	80	Culture-proven infections	Broth microdilution	6
Lombardi [68]	2010–2013	Italy	Prevalence, single center	IRCCS Hospital of San Donato	Various	280	98	NA	Broth microdilution	5
Alicino [69]	2007–2014	Italy	Prevalence, single center	Teaching hospital with 1300 beds	Blood	511	349	Infection occurring 48 h after admission	Broth microdilution	5
Cubero [70]	2010–2012	Spain	Prevalence, single center	Teaching hospital with 900 beds	Various	42	28	CDC/NHSN criteria	Broth microdilution	6
Brizendine [71]	2006–2012	USA	Prevalence, single center	Teaching hospital with 1400 beds	Urine	108	22	CDC/NHSN criteria	Broth microdilution	7
Pouch [72]	2007–2010	USA	Prevalence, single center	2 large academic medical centers in New York City	Urine	272	20	Culture-proven infections	Broth microdilution	5
Japoni-Nejad [73]	2011	Iran	Prevalence, single center	Valiasr Hospital	Various	100	12	NA	Broth microdilution	3
Nobari [74]	2009–2012	Iran	Prevalence, multicenter	8 hospitals in Tehran	Various	180	42	NA	Disc diffusion	5
Simkins [75]	2006–2010	USA	Prevalence, single center	Teaching hospital with 790 beds	Various	52	13	CDC/NHSN criteria	Broth microdilution	6
Rastegar-Lari [76]	2011	Iran	Prevalence, single center	Motahari Burn hospital	Various	35	19	NA	Disc diffusion	2
Kaiser [77]	2007–2009	USA	Prevalence, multicenter	42 US medical centers	Various	2049	126	NA	Broth microdilution	4
Giani [78]	2011	Italy	Prevalence, multicenter	25 large clinical microbiology laboratories	Various	1346	219	NA	Broth microdilution	6
Hussein [79]	2006–2008	Israel	Incidence, single center	A 900-bed tertiary care hospital	Blood	317	103	Infection occurring 48 h after admission	Disc diffusion	7
Mouloudi [80]	2007–2008	Greece	Incidence, single center	A 1000-bed tertiary care hospital	Blood	59	37	Infection occurring 48 h after admission	Broth microdilution	5
Hussein [81]	2006–2007	Israel	Prevalence, single center	A 900-bed tertiary care hospital	Various	461	88	Infection occurring 48 h after admission	Broth microdilution	7
Gasink [82]	2006–2008	USA	Prevalence, single center	A 725-bed tertiary care hospital	Various	928	65	Infection occurring 48 h after admission	Disc diffusion	7
Schwaber [83]	2003–2006	Israel	Prevalence, single center	Teaching hospital with 1200 beds	Various	104	48	Infection occurring 48 h after admission	Disc diffusion	6

Abbreviations: CDC, Centers for Disease Control and Prevention; ECDC, European Centre for Disease Prevention and Control; HAI, hospital-acquired infection; ICU, intensive care unit; KP, *Klebsiella pneumoniae*; LTACH, long-term acute care hospital; NA, not available; NHSN, National Healthcare Safety Network.

**Figure 2.**
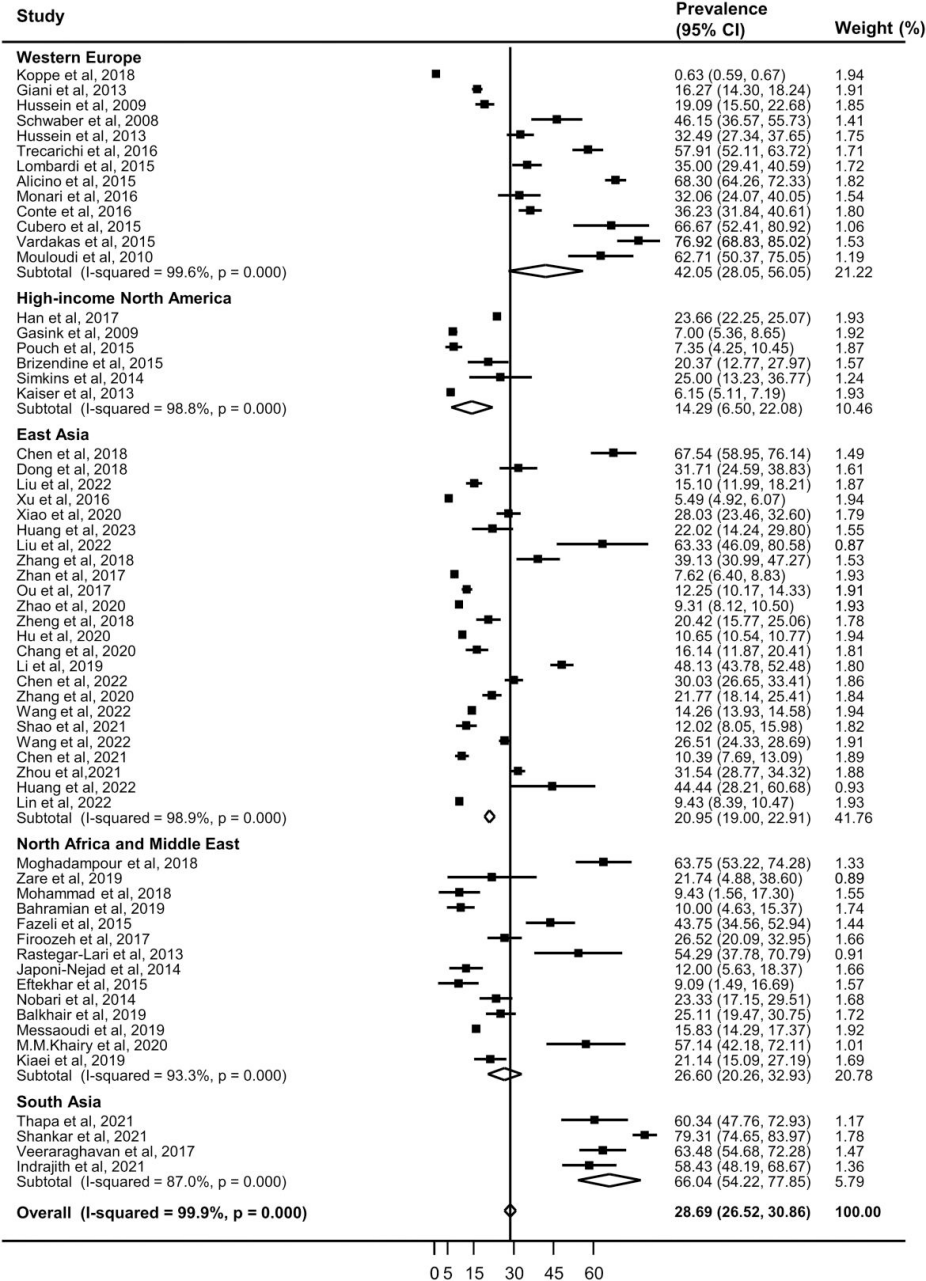
Random-effects meta-analysis: prevalence of hospital-acquired carbapenem-resistant *Klebsiella pneumoniae* infection.

The overall CRKP infection prevalence was estimated to be 28.69% (95% CI, 26.53%–30.86%; 43 500/513 307; Table 2). Considerable heterogeneity was found among studies involved in the analysis (*I*^2^ = 99.8% for the overall population). The estimated pooled CRKP infection prevalence was 20.95% (95% CI, 18.99%–22.91%; 38 940/343 505) in East Asia, 14.29% (95% CI, 6.50%–22.08%; 1067/6879) in high-income North America, 26.60% (95% CI, 20.26%–32.93%; 708/3543) in North Africa and Middle East, 66.04% (95% CI, 54.22%–77.85%; 390/552) in South Asia, and 42.05% (95% CI, 28.05%–50.05%; 2395/158 828) in Western Europe. Notably, patients in South Asia had the highest rates of CRKP infection (Table 2, Figure 2). In SDI level, the pooled CRKP infection prevalence was 18.51% (95% CI, 11.35%–25.68%; 2119/161 727) in high SDI countries, which was much lower than the low and middle SDI countries, which had a prevalence of 29.77% (95% CI, 27.78%–31.75%; 41 381/351 580). No significant difference was found in the prevalence between the methods of antimicrobial susceptibility tests (30.75% for disc diffusion vs 28.03% for broth microdilution).

**Table 2.** Subgroup Prevalence of Carbapenem-Resistant *Klebsiella pneumoniae* Infection

					Univariate Meta-regression		Multivariate Meta-regression	
	Prevalence of CRKP Infection (95% CI)	No. of Participants (No. of Studies)	*I* ^2^, %	*P* Value for Heterogeneity	Coefficient (95% CI)	*P* Value	Coefficient (95% CI)	*P* Value
Overall results	28.69 (26.53–30.86)	513 307 (61)	99.8	<.001	…		…	
Study period					0.03 (–.28 to .35)	.839	0.15 (–.28 to .59)	.479
During or before 2010	30.13 (21.61–38.66)	4346 (9)	98.6	<.0001				
2011–2015	31.05 (25.27–36.84)	19 144 (24)	99.1	<.0001				
During or after 2016	27.44 (24.38–30.51)	489 817 (28)	99.9	<.0001				
Region					2.75 (2.48 to 3.03)	<.001	0.30 (.04 to .56)	.028
East Asia	20.95 (18.99–22.91)	343 505 (24)	98.8	<.0001				
High-income North America	14.29 (6.50–22.08)	6879 (6)	90.5	<.0001				
North Africa and Middle East	26.60 (20.26–32.93)	3543 (14)	93.3	<.0001				
South Asia	66.04 (54.22–77.85)^a^	552 (4)	87.0	<.0001				
Western Europe	42.05 (28.05–50.05)	158 828 (13)	99.6	<.0001				
SDI level					0.89 (–1.50 to –.29)	.004	0.55 (–5.11 to 6.21)	.841
High	18.51 (11.35–25.68)	161 727 (53)	99.5	<.0001				
Low and middle	29.77 (27.78–31.75)^a^	351 580 (8)	99.0	<.0001				
Susceptibility test methods					–0.05 (–.52 to .41)	.820	0.28 (–.21 to .78)	.245
Disc diffusion	30.75 (24.60–36.90)	11 037 (38)	98.7	<.0001				
Broth microdilution	28.03 (25.38–30.70)	502 270 (23)	99.9	<.0001				
Patient cohort					1.00 (–.24 to 2.24)	.112	0.53 (–.71 to 1.78)	.385
ICU	62.31 (34.10–90.53)^a^	11 037 (2)	97.3	<.0001				
Mixed	27.54 (25.35–29.73)	512 696 (611)	99.9	<.0001				
Study design					0.41 (–.09 to .91)	.111	NA^b^	NA^b^
Single center	31.11 (27.34–34.89)	21 496 (45)	91.5	<.0001				
Multicenter	25.65 (21.70–29.61)	491 811 (16)	99.9	<.0001				
Sample source					0.32 (.02 to .63)	.04	0.14 (–.19 to .46)	.397
Blood	34.40 (23.98–44.83)	10 988 (16)	99.3	<.0001				
Urine	11.79 (5.22–18.36)	500 (3)	79.5	.008				
Cerebrospinal fluid	44.44 (28.21–60.68)	36 (1)	NA	NA				
Lower respiratory tract secretions	12.02 (8.05–15.98)	258 (1)	NA	NA				
Various	28.18 (25.53–30.84)	501 525 (40)	99.9	<.0001				
Participant cohort size					–0.84 (–1.28 to –.40)	<.001	–0.29 (–.75 to .16)	.196
<500	36.04 (30.04–42.03)^a^	7385 (42)	97.5	<.0001				
≥500	18.52 (15.09–21.95)	505 922 (19)	100	<.0001				

All subgroup analyses were performed with random-effects meta-analyses.

Abbreviations: CRKP, carbapenem-resistant *Klebsiella pneumoniae*; ICU, intensive care unit; NA, not available; SDI, social development index.

^a^Statistically significant difference.

^b^Study was omitted because of collinearity.

**Figure 3.**
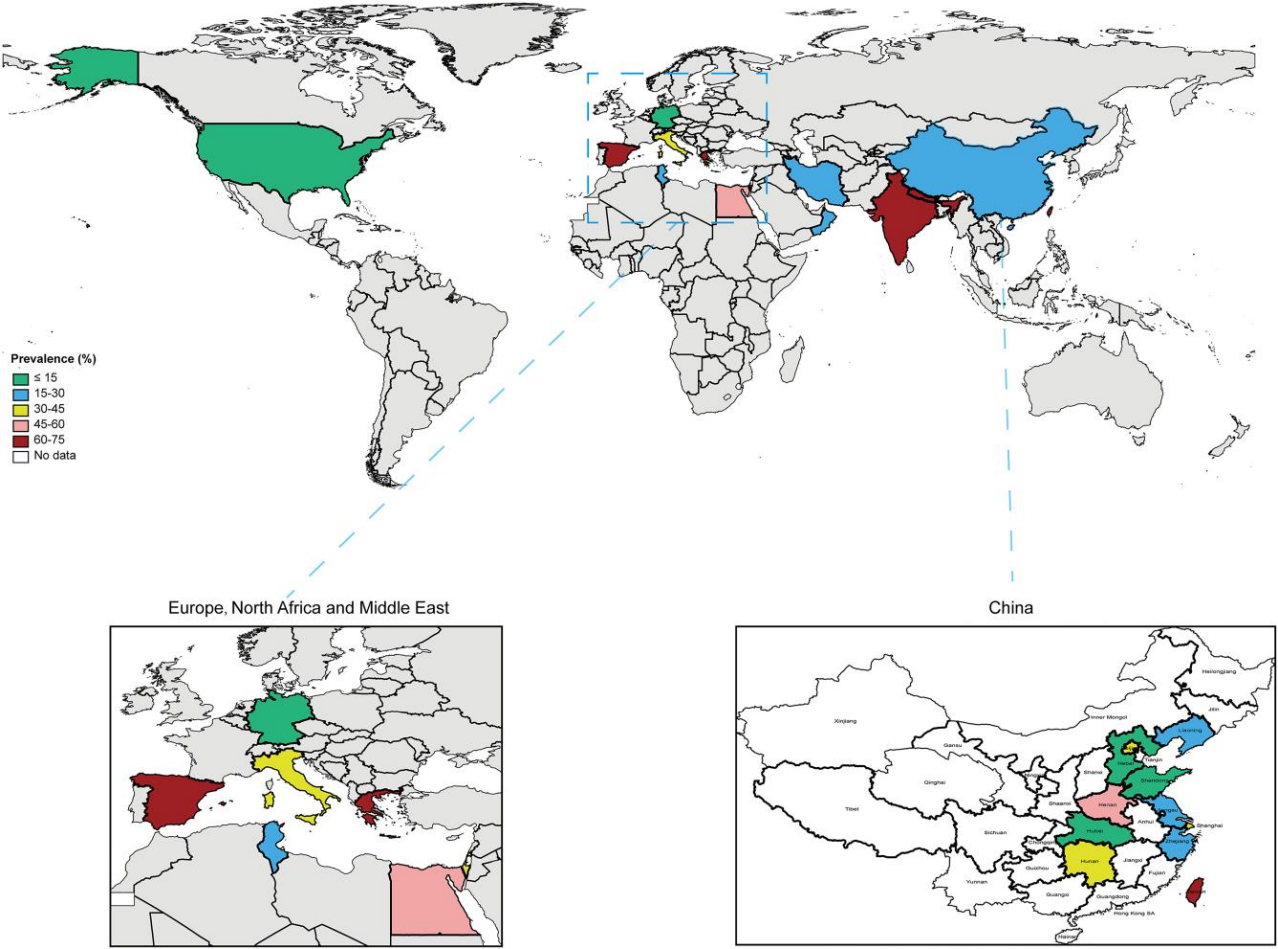
Worldwide geographic distribution: prevalence of hospital-acquired carbapenem-resistant *Klebsiella pneumoniae* infection.

The geographic distributions of the prevalence of CRKP infection are shown in Figure 3 and Supplementary Table 5. Globally, Greece had the highest prevalence at 70.61% (95% CI, 56.77%–84.45%; 117/163), followed by India with 67.62% (95% CI, 53.74%–81.79%; 355/494), and Taiwan with 67.54% (95% CI, 58.65%–76.14%; 77/114). Four countries had a rate of CRKP infection <20% (Germany, United States, Tunisia, and mainland China). Ten provinces of mainland China reported the prevalence of CRKP infection: the highest rates were observed in Henan (48.13%; 95% CI, 43.78%–52.48%), Shanghai (31.55%; 95% CI, 28.78%–34.32%), and Beijing (30.73%; 95% CI, 15.91%–45.55%). Shandong, Hebei, Hubei, Liaoning, Zhejiang, and Jiangsu reported a relatively low rate of CRKP infection, which was <20%.

**Figure 4.**
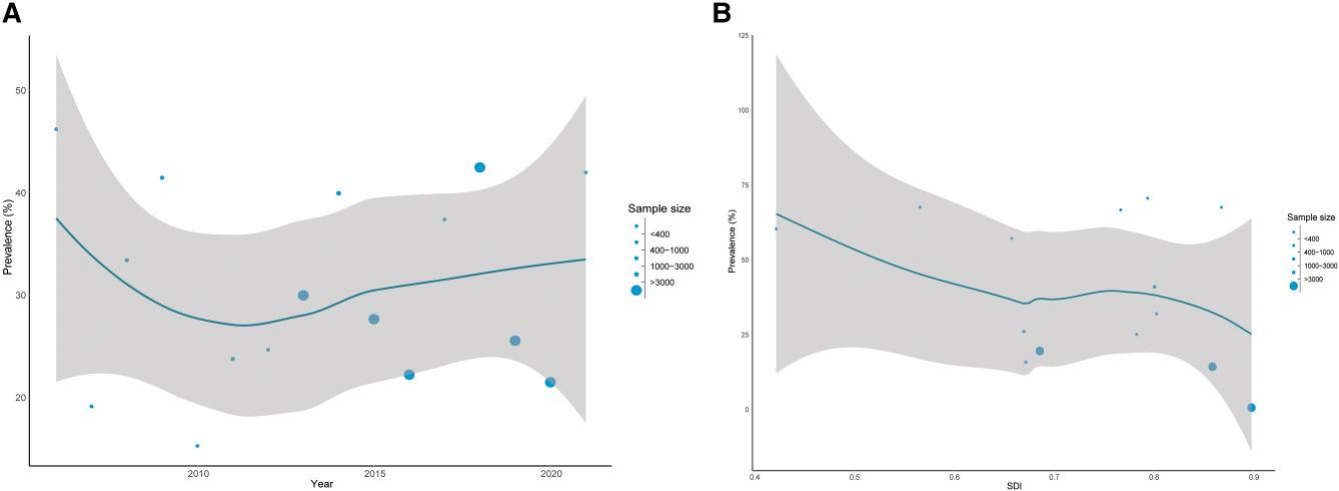
Trends of carbapenem-resistant *Klebsiella pneumoniae* infection: *A*, year; *B*, social development index (SDI). Bubbles are informative data points from studies that reported the prevalence of carbapenem-resistant *Klebsiella pneumoniae*; the size of each bubble is proportional to the sample size. Shaded areas represent 95% CI.

**Figure 5.**
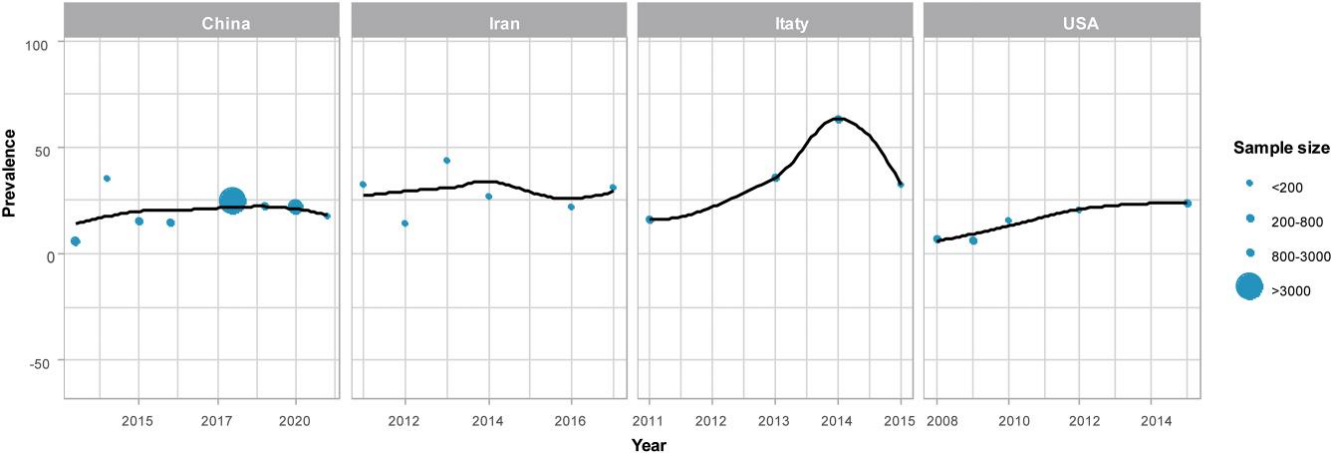
Temporal trends of carbapenem-resistant *Klebsiella pneumoniae* infection in China, Iran, Italy, and the United States. Bubbles are informative data points from studies that reported the prevalence of CRKP; the size of each bubble is proportional to the sample size.

As shown in Figure 4*A*, the pooled CRKP prevalence declined from 2006 to 2010 and then showed an upward trend after 2010. Four countries (China, Iran, Italy, and United States) had >6 publications that reported CRKP infection prevalence, which revealed the changes of CRKP prevalence for these countries. As shown, the upward trend was mainly attributed to Italy and the United States (Figure 5). The relationship between SDI level and CRKP infection prevalence is illustrated in Figure 4*B*. As shown, CRKP infection prevalence was relatively high in countries/territories with a low level of SDI; then, a downward trend occurred until an SDI of about 0.7, when it started to reach a platform.

Multivariate random-effects meta-regression analysis showed that study location (*P* = .028) might be the source of heterogeneity. However, no significant differences were observed in other variables: study period (*P* = .479), SDI level (*P* = .841), or antimicrobial susceptibility test methods (*P* = .245; Table 2). Funnel plot analysis showed evidence of publication bias (Egger test, *P* = .001; Supplementary Figure 1). Sensitivity analysis by excluding any single study did not significantly change the magnitude of the summary effect of the pooled analysis (Supplementary Figure 2).

Due to data availability across studies, 17 associated factors for CRKP infection were evaluated in the meta-analysis. The following were all revealed to be significantly associated with higher odds of CRKP infection: hematologic malignancies, corticosteroid therapies, intensive care unit (ICU) stays, mechanical ventilations, central venous catheter implantations, previous hospitalization, and related antibiotic exposures (eg, antifungals, carbapenems, quinolones, and cephalosporins). However, age was negatively associated with CRKP infection (Table 3).

**Table 3.** Synthesized Effect Size of the Associated Factors of Carbapenem-Resistant *Klebsiella pneumoniae* Infection Investigated in at Least 2 Studies Using Multivariable Logistic Regression

Associated Factor	No. of Studies	Meta-OR (95% CI)	*I* ^2^, %	*P* Value for *Q* Test
Demographics				
Age	2	0.97 (.95–.99)	0	.77
Male sex	2	0.64 (.08–5.25)	82.1	.018
Underlying condition				
Diabetes	4	0.99 (.29–3.32)	73.9	.009
Chronic lung disease	2	0.85 (.40–1.81)	56.7	.128
Hematologic malignancies	2	4.69 (2.80–7.88)	0	.99
Corticosteroids therapy	3	1.31 (1.17–2.21)	0	.739
Exposure to the hospital environment				
ICU stay	8	3.23 (1.59–6.57)	92.7	<.001
Mechanical ventilation	4	2.12 (1.06–4.22)	90.1	<.001
Central venous catheter	3	1.87 (1.16–3.02)	21.1	.282
Previous hospitalization	4	1.03 (1.01–1.06)	0	.516
Source of culture-positive sample				
Blood	4	1.04 (.78–1.39)	57.1	.072
Antibiotic exposure				
Antifungals	2	3.63 (2.07–6.35)	0	.372
Carbapenems	5	3.36 (2.45–4.60)	0	.452
Quinolones	5	2.49 (1.82–3.39)	0	.466
Aminoglycosides	3	1.70 (.65–4.47)	62.3	.07
Cephalosporins	4	2.03 (1.44–2.84)	15.7	.313
Macrolides	2	1.99 (.85–4.66)	55.8	.132

Abbreviations: ICU, intensive care unit; OR, odds ratio.

## DISCUSSION

Globally, hospital-acquired infections caused by KP, particularly CRKP strains, pose a significant threat to patients’ safety due to their scarce treatment options. This systematic review and meta-analysis provided a comprehensive summary of recent data on the prevalence of nosocomial infections caused by CRKP in 5 GBD-defined regions: East Asia, high-income North America, North Africa and Middle East, South Asia, and Western Europe. In this study, epidemiologic data were extracted on CRKP prevalence and associated risk factors spread over 2 decades (2003–2021), approximately involving 43 500 CRKP infections among 513 307 nosocomial KP infections from 14 countries/territories. The pooled CRKP prevalence was estimated to be 28.69% globally, with hematologic malignancies having the highest odds ratio leaving patients prone to CRKP infection. Collectively, these findings demonstrated a significant global burden of CRKP infection.

Subsequent to the study by Tesfad et al that quantitatively estimated the epidemiology of CRKP colonization [13], this study made the first attempt of estimating the global CRKP infection prevalence. By far, this study provides the most up-to-date estimation of CRKP prevalence at the global level and summarizes the risk factors for CRKP infection in hospital-acquired KP infections.

Although nosocomial CRKP infections pose a global threat, characteristics of the CRKP epidemics vary a lot by country and region. For instance, in the GBD region level, the prevalence of CRKP in South Asia was 66.04%, which was much higher than in other regions. Particularly, KP was the most common multidrug-resistant pathogen isolated in Indian hospitals and is related to several outbreaks [84–86]. The highly mosaic genome of KP coupled with its diverse ecologic niches probably accounted for the relatively higher proportion of CRKP infections in India as compared with other countries [32]. CRKP infection prevalence was reported in 23 publications from mainland China (related to 10 provinces), with a pooled prevalence of 19.60% (95% CI, 17.67%–21.49%). The narrow 95% CI around the effect estimation indicates the reliability of the pooled analysis. The distribution of CRKP infection rates ranged from 12.02% in Shandong to 48.13% in Henan, demonstrating a substantial regional difference in CRKP burden in China. Data from the 2021 China Antimicrobial Surveillance Network (http://www.chinets.com/) had corroborated our findings, showing a CRKP rate of 50.8% in Henan vs 14.4% in Shandong, which implied that much more targeted strategies should be implemented in some provinces (eg, Henan) to predict the increase in CRKP infection.

Importantly, hospital-acquired CRKP infection prevalence is particularly high in ICUs, where the pooled estimate is as high as 62.31%. ICUs are notorious hotbeds for hospital-acquired infections, including CRKP, because of vulnerable patients with several invasive procedures, catheter placements, and severe comorbidities [87, 88]. Furthermore, the prolonged stay of patients in the ICU may act as a reservoir for CRKP and promote transmission among patients who are critically ill. In this study, studies from China [44] and Greece [67] reported the prevalence of CRKP infection in the ICU, and no systematic differences were found between them. To reduce the CRKP prevalence, rigorous infection controls should be implemented in the ICU, such as active surveillance culture, disinfection, initial appropriate antimicrobial therapy, and a timely antibiotic de-escalation strategy. It is important to note that our pooled analysis on the ICU might be overrepresented by the 2 countries; hence, further investigations are necessary.

Consistent with previous reports, hematologic malignancies were the principal risk factor related to CRKP infection, with a risk approximately 4.7 times higher vs no hematologic malignancies [5, 89]. Zhang et al demonstrated that the main risk factor for the development of CRKP bloodstream infection was hematologic malignancy [53]. The widespread development of CRKP in hematologic settings might be attributed to well-known risk factors for infections, such as more invasive procedures, long-term hospitalization, and being exposed to high-grade antibiotics. Furthermore, the prolonged postchemotherapy neutropenia particularly impairs immunologic response and predisposes these patients to severe infections [90].

In our analysis, several antibiotic administrations were associated with the development of CRKP infections: carbapenems, antifungals, quinolones, and cephalosporins. Antibiotic selective pressure is well known to mainly cause drug-resistant strain infections. Previous evidence confirmed that using carbapenem antibiotics could lead to KP carbapenemase production [91]. Additionally, Kritsotakis et al found that increased exposure to one antibiotic group enhances the risk of KP resistance to another antibiotic group [92]. Therefore, combined antibiotic therapy and longer treatment courses increased the antibiotic selection pressure, enabling carbapenem-resistant strains to develop a plethora of resistance mechanisms [93].

### Strengths and Limitations

Benefiting from comprehensive systematic review and a rigorous selection criterion, our ability to obtain relevant literature on CRKP epidemiology could be well guaranteed. To compute the prevalence of CRKP infection, 61 individual studies were ultimately included for pooled analysis. Only studies that were performed in cases of general in-hospital KP infection were included, which could largely ensure the generalizability of our estimation on CRKP epidemiology. Regarding the risk factors for CRKP infection, only the studies that were based on a multivariate design were included to reduce potential bias [94].

This study also has several limitations. First, interstudy heterogeneity and publication bias were detected. Thus, the pooled effect size of CRKP prevalence must be interpreted with caution, emphasizing the need for more robust surveillance of CRKP infection. Subgroup analyses could not explain the specific causes of heterogeneity (*I*^2^ > 50%, *P* < .001), which might be from various factors, such as study period, geographic distribution, SDI level, sample size, sample source, or susceptibility test methods. Meta-regression analysis was performed on various sources, and significant differences in CRKP infection were observed among different regions (Table 2), implying that this might be the main cause of heterogeneity in this meta-analysis. Second, several potentially eligible studies were included through our systematic review, but a risk remains that we missed some relevant data. Third, the country representativeness of various studies is unclear, which to some extent limits the external validity of our findings. Third, investigations are not distributed evenly across the GBD regions. Fourth, for research that took several years, the last year was used to estimate the point prevalence, which might not precisely reflect the temporal trend. Last, in some parts of the world, CRKP rates may be underestimated depending on the version of minimal inhibitory concentration breakpoints being used. Carbapenem breakpoint changes that occurred in 2010 contributed to increased reporting of carbapenem-resistant Enterobacteriaceae, but several laboratories are behind in adopting these changes and the use of breakpoints varies among them. This is particularly true in the United States, where adoption of 2010 breakpoint changes has been slow due to various factors.

## CONCLUSION

The results of our global meta-analysis show that patients with nosocomial KP infection have a high rate of CRKP infection, especially in South Asia or low SDI countries. This study also reaffirms the importance of hematologic malignancies, ICU stays, and antibiotic exposures as the leading risks of CRKP infection globally. Although data remain sparse in many countries, our research has collected the most up-to-date data from available publications on CRKP prevalence and associated risk factors, which can help shape the global response to infection prevention and control.

## Supplementary Material

ofad649_Supplementary_Data

## Supplementary Data

Supplementary materials are available at *Open Forum Infectious Diseases* online. Consisting of data provided by the authors to benefit the reader, the posted materials are not copyedited and are the sole responsibility of the authors, so questions or comments should be addressed to the corresponding author.

## Notes

***Financial support*.** This work was supported by the National Natural Science Foundation of China (82000479); and the China Postdoctoral Science Foundation (2020M682422).
