# Supplementary material for: The Global and Regional Prevalence of Hospital-Acquired Carbapenem-Resistant Klebsiella pneumoniae Infection: A Systematic Review and Meta-analysis
Source: Open Forum Infect Dis. 2023 Dec 19;11(2):ofad649. doi: 10.1093/ofid/ofad649 (PMC10836986; doi:10.1093/ofid/ofad649)

**Table S1** Search strategy

Source: PubMed; Searched on: March 30, 2023; Results: 2644

| Search | Query | Results |
| --- | --- | --- |
| 1 | Klebsiella pneumoniae | 17247 |
| 2 | K. pneumoniae | 17,853 |
| 3 | #1 OR #2 | 17853 |
| 4 | carbapenem | 16903 |
| 5 | imipenem | 8657 |
| 6 | meropenem | 6502 |
| 7 | ertapenem | 1462 |
| 8 | #4 OR #5OR #6 OR #7 | 22452 |
| 9 | resistance | 754905 |
| 10 | #8 AND #9 | 16,413 |
| 11 | #3 AND #4 AND #10 | 4540 |
| 12 | (((prevalence) OR (epidemiological data)) OR (epidemiolog*)) OR (frequenc*) | 3,699,686 |
| 13 | #11 AND #12 | 2644 |

Source: Embase; Searched on: March 30, 2023; Results: 835

| Search | Query | Results |
| --- | --- | --- |
| 1 | 'carbapenem resistant klebsiella pneumoniae'/exp OR 'carbapenem resistant klebsiella pneumoniae' | 2135 |
| 2 | prevalence | 1,343,327 |
| 3 | epidemiolog* | 2,549,625 |
| 4 | frequenc* | 1,562,397 |
| 5 | #2 OR #3 OR #4 | 4,733,585 |
| 6 | #1 AND #5 | 835 |

Source: Medline; Searched on: March 30, 2023; Results: 2508

| Search | Query | Results |
| --- | --- | --- |
| 1 | (TS=(Klebsiella pneumoniae)) OR TS=(K. pneumoniae) | 33390 |
| 2 | (((TS=(carbapenem)) OR TS=(imipenem)) OR TS=(meropenem)) OR TS=(ertapenem) | 30350 |
| 3 | (#2) AND TS=(resistance) | 19019 |
| 4 | (((TS=(prevalence)) OR TS=(epidemiological data)) OR TS=(epidemiolog*)) OR TS=(frequenc*) | 3680437 |
| 5 | #1 and #3 and #4 | 2508 |

**Table S2** Quality assessment scale for rating the risk of bias

| **Bias type** | **Low risk (score=2)** | **Moderate risk (score=1)** | **High risk (score=0)** |
| --- | --- | --- | --- |
| Sample population | 1. Sample from the general population, not a select group; 2. Consecutive unselected population; 3. Rationale for case and control selection explained. | 1. Sample selected from large population but selection criteria not defined; 2. Sample selection ambiguous but may be representative; 3. Rationale for cases and controls not explained; 4. Eligibility criteria not explained; 5. Analysis to adjust for sampling strategy bias. | 1. Highly select population making it difficult to generalize finding; 2. Sample selection ambiguous and sample unlikely to be representative. |
| Sample size | >200 | 100-200 | <100 |
| Outcome assessment | 1. Diagnosis using consistent criteria and direct examination. | 1. Assessment from administrative database or register; 2. Assessment from hospital record or interviewer. | 1. Assessment from non-validated data or generic estimate from the overall population. |
| Analytical methods to control for bias | 1. Analysis appropriate for the type of sample (subgroup analysis/regression etc.). | 1. Analysis does not account for common adjustment. | 1. Data confusing. |

**Table S3** List of the included articles (n=61)

A1. Huang Y, Li J, Wang Q, Tang K, Cai X, Li C. Detection of carbapenem-resistant hypervirulent Klebsiella pneumoniae ST11-K64 co-producing NDM-1 and KPC-2 in a tertiary hospital in Wuhan. J Hosp Infect. 2023; 131:70-80.

A2. Liu N, Yang G, Dang Y, Liu X, Chen M, Dai F*,* et al. Epidemic, risk factors of carbapenem-resistant Klebsiella pneumoniae infection and its effect on the early prognosis of liver transplantation. Front Cell Infect Mi. 2022; 12:976408.

A3. Huang N, Jia H, Zhou B, Zhou C, Cao J, Liao W*,* et al. Hypervirulent carbapenem-resistant Klebsiella pneumoniae causing highly fatal meningitis in southeastern China. Front Public Health. 2022; 10:991306.

A4. Chen J, Ma H, Huang X, Cui Y, Peng W, Zhu F*,* et al. Risk factors and mortality of carbapenem-resistant Klebsiella pneumoniae bloodstream infection in a tertiary-care hospital in China: an eight-year retrospective study. Antimicrob Resist in. 2022; 11:161.

A5. Wang Q, Chen M, Ou Q, Zheng L, Chen X, Mao G*,* et al. Carbapenem-resistant hypermucoviscous Klebsiella pneumoniae clinical isolates from a tertiary hospital in China: Antimicrobial susceptibility, resistance phenotype, epidemiological characteristics, microbial virulence, and risk factors. Front Cell Infect Mi. 2022; 12:1083009.

A6. Liu C, Liu L, Jin MM, Hu YB, Cai X, Wan L*,* et al. Molecular Epidemiology and Risk Factors of Carbapenem-Resistant Klebsiella Pneumoniae Bloodstream Infections in Wuhan, China. Curr Med Sci. 2022; 42:68-76.

A7. Lin Z, Yu J, Liu S, Zhu M. Prevalence and antibiotic resistance of Klebsiella pneumoniae in a tertiary hospital in Hangzhou, China, 2006–2020. J Int Med Res. 2022; 50:1410680941.

A8. Wang N, Zhan M, Liu J, Wang Y, Hou Y, Li C*,* et al. Prevalence of Carbapenem-Resistant Klebsiella pneumoniae Infection in a Northern Province in China: Clinical Characteristics, Drug Resistance, and Geographic Distribution. Infect Drug Resist. 2022; 15:569-79.

A9. Indrajith S, Mukhopadhyay AK, Chowdhury G, Farraj D, Alkufeidy RM, Natesan S*,* et al. Molecular insights of Carbapenem resistance Klebsiella pneumoniae isolates with focus on multidrug resistance from clinical samples. J Infect Public Heal. 2021; 14:131-8.

A10. Shankar C, Jacob JJ, Sugumar SG, Natarajan L, Rodrigues C, Mathur P*,* et al. Distinctive Mobile Genetic Elements Observed in the Clonal Expansion of Carbapenem-Resistant Klebsiella pneumoniae in India. Microb Drug Resist. 2021; 27:1096-104.

A11. Shao C, Wang W, Liu S, Zhang Z, Jiang M, Zhang F. Molecular Epidemiology and Drug Resistant Mechanism of Carbapenem-Resistant Klebsiella pneumoniae in Elderly Patients With Lower Respiratory Tract Infection. Front Public Health. 2021; 9:669173.

A12. Thapa S, Adhikari N, Shah AK, Lamichhane I, Dhungel B, Shrestha UT*,* et al. Detection of NDM-1 and VIM Genes in Carbapenem-Resistant Klebsiella pneumoniae Isolates from a Tertiary Health-Care Center in Kathmandu, Nepal. Chemotherapy. 2021; 66:199-209.

A13. Chen J, Hu C, Wang R, Li F, Sun G, Yang M, et al. Shift in the Dominant Sequence Type of Carbapenem-Resistant Klebsiella pneumoniae Bloodstream Infection from ST11 to ST15 at a Medical Center in Northeast China, 2015-2020. Infect Drug Resist. 2021; 14:1855-63.

A14. Zhou C, Wu Q, He L, Zhang H, Xu M, Yuan B*,* et al. Clinical and Molecular Characteristics of Carbapenem-Resistant Hypervirulent Klebsiella pneumoniae Isolates in a Tertiary Hospital in Shanghai, China. Infect Drug Resist. 2021; 14:2697-706.

A15. Khairy R, Mahmoud MS, Shady RR, Esmail M. Multidrug-resistant Klebsiella pneumoniae in hospital-acquired infections: Concomitant analysis of antimicrobial resistant strains. Int J Clin Pract. 2020; 74:e13463.

A16. Chang H, Wei J, Zhou W, Yan X, Cao X, Zuo L*,* et al. Risk factors and mortality for patients with Bloodstream infections of Klebsiella pneumoniae during 2014-2018: Clinical impact of carbapenem resistance in a large tertiary hospital of China. J Infect Public Heal. 2020; 13:784-90.

A17. Xiao T, Zhu Y, Zhang S, Wang Y, Shen P, Zhou Y*,* et al. A Retrospective Analysis of Risk Factors and Outcomes of Carbapenem-Resistant Klebsiella pneumoniae Bacteremia in Nontransplant Patients. J Infect Dis. 2020; 221:S174-83.

A18.Zhang G, Zhang M, Sun F, Zhou J, Wang Y, Zhu D*,* et al. Epidemiology, mortality and risk factors for patients with K. pneumoniae bloodstream infections: Clinical impact of carbapenem resistance in a tertiary university teaching hospital of Beijing. J Infect Public Heal. 2020; 13:1710-4.

A19. Hu Y, Liu C, Shen Z, Zhou H, Cao J, Chen S*,* et al. Prevalence, risk factors and molecular epidemiology of carbapenem-resistant Klebsiella pneumoniae in patients from Zhejiang, China, 2008-2018. Emerg Microbes Infec. 2020; 9:1771-9.

A20. Zhao Y, Zhang S, Fang R, Wu Q, Li J, Zhang Y*,* et al. Dynamic Epidemiology and Virulence Characteristics of Carbapenem-Resistant Klebsiella pneumoniae in Wenzhou, China from 2003 to 2016. Infect Drug Resist. 2020; 13:931-40.

A21. Kiaei S, Moradi M, Hosseini-Nave H, Ziasistani M, Kalantar-Neyestanaki D. Endemic dissemination of different sequence types of carbapenem-resistant Klebsiella pneumoniae strains harboring bla (NDM) and 16S rRNA methylase genes in Kerman hospitals, Iran, from 2015 to 2017. Infect Drug Resist. 2019; 12:45-54.

A22. Li Y, Shen H, Zhu C, Yu Y. Carbapenem-Resistant Klebsiella pneumoniae Infections among ICU Admission Patients in Central China: Prevalence and Prediction Model. Biomed Res Int. 2019; 2019:9767313.

A23. Balkhair A, Al-Muharrmi Z, Al'Adawi B, Al BI, Taher HB, Al-Siyabi T*,* et al. Prevalence and 30-day all-cause mortality of carbapenem-and colistin-resistant bacteraemia caused by Acinetobacter baumannii, Pseudomonas aeruginosa, and Klebsiella pneumoniae: Description of a decade-long trend. Int J Infect Dis. 2019; 85:10-5.

A24. Messaoudi A, Mansour W, Jaidane N, Chaouch C, Boujaâfar N, Bouallègue O. Epidemiology of resistance and phenotypic characterization of carbapenem resistance mechanisms in Klebsiella pneumoniae isolates at Sahloul University Hospital-Sousse, Tunisia. Afr Health Sci. 2019; 19:2008-20.

A25. Bahramian A, Shariati A, Azimi T, Sharahi JY, Bostanghadiri N, Gachkar L*,* et al. First report of New Delhi metallo-β-lactamase-6 (NDM-6) among Klebsiella pneumoniae ST147 strains isolated from dialysis patients in Iran. Infection, Genetics and Evolution. 2019; 69:142-5.

A26. Zare D, Fazeli H. First prevalence of metallo beta-lactamases producing Enterobacteriacea in Iranian cancer patients. Annali di igiene. 2019; 31:62.

A27. Mohammad Ali Tabrizi A, Badmasti F, Shahcheraghi F, Azizi O. Outbreak of hypervirulent Klebsiella pneumoniae harbouring blaVIM-2 among mechanically-ventilated drug-poisoning patients with high mortality rate in Iran. J Glob Antimicrob Re. 2018; 15:93-8.

A28. Dong F, Zhang Y, Yao K, Lu J, Guo L, Lyu S*,* et al. Epidemiology of Carbapenem-Resistant Klebsiella pneumoniae Bloodstream Infections in a Chinese Children's Hospital: Predominance of New Delhi Metallo-β-Lactamase-1. Microb Drug Resist. 2018; 24:154-60.

A29. Zheng SH, Cao SJ, Xu H, Feng D, Wan LP, Wang GJ*,* et al. Risk factors, outcomes and genotypes of carbapenem-nonsusceptible Klebsiella pneumoniae bloodstream infection: a three-year retrospective study in a large tertiary hospital in Northern China. Infect Dis-Nor. 2018; 50:443-51.

A30. Chen JY, Liou ML, Kuo HY, Lu CW, Lai YC, Lin YY*,* et al. Dissemination of carbapenem-resistant Klebsiella pneumoniae harboring KPC-carrying plasmid pKPC_P16, a pKPC_LK30 variant, in northern Taiwan. Diagn Micr Infec Dis. 2018; 91:291-3.

A31. Zhang Y, Guo LY, Song WQ, Wang Y, Dong F, Liu G. Risk factors for carbapenem-resistant K. pneumoniae bloodstream infection and predictors of mortality in Chinese paediatric patients. Bmc Infect Dis. 2018; 18:248.

A32. Koppe U, von Laer A, Kroll LE, Noll I, Feig M, Schneider M*,* et al. Carbapenem non-susceptibility of Klebsiella pneumoniae isolates in hospitals from 2011 to 2016, data from the German Antimicrobial Resistance Surveillance (ARS). Antimicrob Resist in. 2018; 7:71.

A33. Moghadampour M, Rezaei A, Faghri J. The emergence of bla(OXA-48) and bla(NDM) among ESBL-producing Klebsiella pneumoniae in clinical isolates of a tertiary hospital in Iran. Acta Microbiol Imm H. 2018; 65:335-44.

A34. Firoozeh F, Mahluji Z, Shams E, Khorshidi A, Zibaei M. New Delhi metallo-β-lactamase-1-producing Klebsiella pneumoniae isolates in hospitalized patients in Kashan, Iran. Iran J Microbiol. 2017; 9:283-7.

A35. Han JH, Goldstein EJ, Wise J, Bilker WB, Tolomeo P, Lautenbach E. Epidemiology of Carbapenem-Resistant Klebsiella pneumoniae in a Network of Long-Term Acute Care Hospitals. Clin Infect Dis. 2017; 64:839-44.

A36. Zhan L, Wang S, Guo Y, Jin Y, Duan J, Hao Z*,* et al. Outbreak by Hypermucoviscous Klebsiella pneumoniae ST11 Isolates with Carbapenem Resistance in a Tertiary Hospital in China. Front Cell Infect Mi. 2017; 7:182.

A37. Veeraraghavan B, Shankar C, Karunasree S, Kumari S, Ravi R, Ralph R. Carbapenem resistant Klebsiella pneumoniae isolated from bloodstream infection: Indian experience. Pathog Glob Health. 2017; 111:240-6.

A38. Ou Q, Li W, Li B, Yu C. Prevalence of Carbapenem-Resistant Klebsiella Pneumoniae (CRKP) and the Distribution of Class 1 Integron in Their Strains Isolated from a Hospital in Central China. Chin Med Sci J. 2017; 32:102-7.

A39. Monari C, Merlini L, Nardelli E, Cacioni M, Repetto A, Mencacci A*,* et al. Carbapenem-Resistant Klebsiella pneumoniae: Results of a Laboratory Surveillance Program in an Italian General Hospital (August 2014-January 2015): Surveillance of Carbapenem-resistant Klebsiella pneumoniae. Adv Exp Med Biol. 2016; 901:91-101.

A40. Xu A, Zheng B, Xu YC, Huang ZG, Zhong NS, Zhuo C. National epidemiology of carbapenem-resistant and extensively drug-resistant Gram-negative bacteria isolated from blood samples in China in 2013. Clin Microbiol Infec. 2016; 22 Suppl 1:S1-8.

A41. Trecarichi EM, Pagano L, Martino B, Candoni A, Di Blasi R, Nadali G*,* et al. Bloodstream infections caused by Klebsiella pneumoniae in onco-hematological patients: clinical impact of carbapenem resistance in a multicentre prospective survey. Am J Hematol. 2016; 91:1076-81.

A42. Conte V, Monaco M, Giani T, D'Ancona F, Moro ML, Arena F*,* et al. Molecular epidemiology of KPC-producingKlebsiella pneumoniae from invasive infections in Italy: increasing diversity with predominance of the ST512 clade II sublineage. J Antimicrob Chemoth. 2016; 71:3386-91.

A43. Eftekhar F, Naseh Z. Extended-spectrum beta-lactamase and carbapenemase production among burn and non-burn clinical isolates of Klebsiella pneumoniae. Iran J Microbiol. 2015; 7:144-9.

A44. Fazeli H, Norouzi-Barough M, Ahadi AM, Shokri D, Solgi H. Detection of New Delhi Metallo-Beta-Lactamase-1 (NDM-1) in carbapenem- resistant Klebsiella pneumoniae isolated from a university hospital in Iran. Hippokratia. 2015; 19:205-9.

A45. Vardakas KZ, Matthaiou DK, Falagas ME, Antypa E, Koteli A, Antoniadou E. Characteristics, risk factors and outcomes of carbapenem-resistant Klebsiella pneumoniae infections in the intensive care unit. J Infection. 2015; 70:592-9.

A46. Lombardi F, Gaia P, Valaperta R, Cornetta M, Tejada MR, Di Girolamo L*,* et al. Emergence of Carbapenem-Resistant Klebsiella pneumoniae: Progressive Spread and Four-Year Period of Observation in a Cardiac Surgery Division. Biomed Res Int. 2015; 2015:871947.

A47. Alicino C, Giacobbe DR, Orsi A, Tassinari F, Trucchi C, Sarteschi G*,* et al. Trends in the annual incidence of carbapenem-resistant Klebsiella pneumoniae bloodstream infections: a 8-year retrospective study in a large teaching hospital in northern Italy. Bmc Infect Dis. 2015; 15:415.

A48. Cubero M, Cuervo G, Dominguez MÁ, Tubau F, Martí S, Sevillano E*,* et al. Carbapenem-resistant and carbapenem-susceptible isogenic isolates of Klebsiella pneumoniae ST101 causing infection in a tertiary hospital. Bmc Microbiol. 2015; 15:177.

A49. Brizendine KD, Richter SS, Cober ED, van Duin D. Carbapenem-resistant Klebsiella pneumoniae urinary tract infection following solid organ transplantation. Antimicrob Agents Ch. 2015; 59:553-7.

A50. Pouch SM, Kubin CJ, Satlin MJ, Tsapepas DS, Lee JR, Dube G*,* et al. Epidemiology and outcomes of carbapenem-resistant Klebsiella pneumoniae bacteriuria in kidney transplant recipients. Transpl Infect Dis. 2015; 17:800-9.

A51. Japoni-Nejad A, Ghaznavi-Rad E, van Belkum A. Characterization of Plasmid-Mediated AmpC and Carbapenemases among Iranain Nosocomial Isolates of Klebsiella pneumoniae Using Phenotyping and Genotyping Methods. Osong Public Health Res Perspect. 2014; 5:333-8.

A52. Nobari S, Shahcheraghi F, Rahmati GF, Valizadeh B. Molecular characterization of carbapenem-resistant strains of Klebsiella pneumoniae isolated from Iranian patients: first identification of blaKPC gene in Iran. Microb Drug Resist. 2014; 20:285-93.

A53. Simkins J, Muggia V, Cohen HW, Minamoto GY. Carbapenem-resistant Klebsiella pneumoniae infections in kidney transplant recipients: a case-control study. Transpl Infect Dis. 2014; 16:775-82.

A54. Rastegar Lari A, Azimi L, Rahbar M, Fallah F, Alaghehbandan R. Phenotypic detection of Klebsiella pneumoniae carbapenemase among burns patients: First report from Iran. Burns. 2013; 39:174-6.

A55. Kaiser RM, Castanheira M, Jones RN, Tenover F, Lynfield R. Trends in Klebsiella pneumoniae carbapenemase-positive K. pneumoniae in US hospitals: report from the 2007-2009 SENTRY Antimicrobial Surveillance Program. Diagn Micr Infec Dis. 2013; 76:356-60.

A56. Giani T, Pini B, Arena F, Conte V, Bracco S, Migliavacca R*,* et al. Epidemic diffusion of KPC carbapenemase-producing Klebsiella pneumoniae in Italy: results of the first countrywide survey, 15 May to 30 June 2011. Eurosurveillance. 2013; 18.

A57. Hussein K, Raz-Pasteur A, Finkelstein R, Neuberger A, Shachor-Meyouhas Y, Oren I*,* et al. Impact of carbapenem resistance on the outcome of patients' hospital-acquired bacteraemia caused by Klebsiella pneumoniae. J Hosp Infect. 2013; 83:307-13.

A58. Mouloudi E, Protonotariou E, Zagorianou A, Iosifidis E, Karapanagiotou A, Giasnetsova T*,* et al. Bloodstream infections caused by metallo-beta-lactamase/Klebsiella pneumoniae carbapenemase-producing K. pneumoniae among intensive care unit patients in Greece: risk factors for infection and impact of type of resistance on outcomes. Infect Cont Hosp Ep. 2010; 31:1250-6.

A59. Hussein K, Sprecher H, Mashiach T, Oren I, Kassis I, Finkelstein R. Carbapenem resistance among Klebsiella pneumoniae isolates: risk factors, molecular characteristics, and susceptibility patterns. Infect Cont Hosp Ep. 2009; 30:666-71.

A60. Gasink LB, Edelstein PH, Lautenbach E, Synnestvedt M, Fishman NO. Risk factors and clinical impact of Klebsiella pneumoniae carbapenemase-producing K. pneumoniae. Infect Cont Hosp Ep. 2009; 30:1180-5.

A61. Schwaber MJ, Klarfeld-Lidji S, Navon-Venezia S, Schwartz D, Leavitt A, Carmeli Y. Predictors of carbapenem-resistant Klebsiella pneumoniae acquisition among hospitalized adults and effect of acquisition on mortality. Antimicrob Agents Ch. 2008; 52:1028-33.

**Table S4** Quality scores for assessing the risk of bias in the included articles (n=61)

| **Study ID** | **Author (publish year)** | **Quality score** | | | | |
| --- | --- | --- | --- | --- | --- | --- |
|  |  | **Sample population** | **Sample size** | **Outcome assessment** | **Analytical methods** | **Total scores** |
| A1 | Huang et al. (2023) | 0 | 1 | 1 | 2 | 4 |
| A2 | Liu et al. (2022) | 0 | 0 | 2 | 2 | 4 |
| A3 | Huang et al. (2022) | 2 | 0 | 1 | 2 | 5 |
| A4 | Chen et al. (2022) | 2 | 2 | 2 | 2 | 8 |
| A5 | Wang et al. (2022) | 2 | 2 | 1 | 2 | 7 |
| A6 | Liu et al. (2022) | 2 | 2 | 2 | 2 | 8 |
| A7 | Lin et al. (2022) | 2 | 2 | 0 | 2 | 6 |
| A8 | Wang et al. (2022) | 2 | 2 | 0 | 2 | 6 |
| A9 | Indrajith et al. (2021) | 2 | 0 | 0 | 2 | 4 |
| A10 | Shankar et al. (2021) | 2 | 2 | 0 | 2 | 6 |
| A11 | Shao et al. (2021) | 2 | 2 | 1 | 2 | 7 |
| A12 | Thapa et al. (2021) | 2 | 0 | 0 | 2 | 4 |
| A13 | Chen et al. (2021) | 2 | 2 | 0 | 2 | 6 |
| A14 | Zhou et al. (2021) | 2 | 2 | 0 | 2 | 6 |
| A15 | M.M.Khairy et al. (2020) | 2 | 0 | 2 | 2 | 6 |
| A16 | Chang et al. (2020) | 2 | 2 | 1 | 2 | 7 |
| A17 | Xiao et al. (2020) | 0 | 2 | 2 | 2 | 6 |
| A18 | Zhang et al. (2020) | 2 | 2 | 1 | 2 | 7 |
| A19 | Hu et al. (2020) | 2 | 2 | 0 | 2 | 6 |
| A20 | Zhao et al. (2020) | 2 | 2 | 0 | 2 | 6 |
| A21 | Kiaei et al. (2019) | 2 | 1 | 1 | 2 | 6 |
| A22 | Li et al. (2019) | 1 | 2 | 1 | 2 | 6 |
| A23 | Balkhair et al. (2019) | 2 | 2 | 1 | 2 | 7 |
| A24 | Messaoudi et al. (2019) | 2 | 2 | 0 | 2 | 6 |
| A25 | Bahramian et al. (2019) | 0 | 1 | 0 | 2 | 3 |
| A26 | Zare et al. (2019) | 2 | 0 | 0 | 2 | 4 |
| A27 | Mohammad et al. (2018) | 0 | 0 | 1 | 2 | 3 |
| A28 | Dong et al. (2018) | 2 | 1 | 1 | 2 | 6 |
| A29 | Zheng et al. (2018) | 2 | 2 | 1 | 2 | 7 |
| A30 | Chen et al. (2018) | 2 | 1 | 0 | 2 | 5 |
| A31 | Zhang et al. (2018) | 2 | 1 | 1 | 2 | 6 |
| A32 | Koppe et al. (2018) | 2 | 2 | 0 | 2 | 6 |
| A33 | Moghadampour et al. (2018) | 2 | 0 | 0 | 2 | 4 |
| A34 | Firoozeh et al. (2017) | 2 | 1 | 0 | 2 | 5 |
| A35 | Han et al. (2017) | 2 | 2 | 2 | 2 | 8 |
| A36 | Zhan et al. (2017) | 2 | 2 | 0 | 2 | 6 |
| A37 | Veeraraghavan et al. (2017) | 2 | 1 | 1 | 2 | 6 |
| A38 | Ou et al. (2017) | 2 | 2 | 0 | 2 | 6 |
| A39 | Monari et al. (2016) | 2 | 1 | 2 | 2 | 7 |
| A40 | Xu et al. (2016) | 2 | 2 | 0 | 2 | 6 |
| A41 | Trecarichi et al. (2016) | 0 | 2 | 1 | 2 | 5 |
| A42 | Conte et al. (2016) | 0 | 2 | 0 | 2 | 4 |
| A43 | Eftekhar et al. (2015) | 2 | 0 | 0 | 2 | 4 |
| A44 | Fazeli et al. (2015) | 2 | 1 | 0 | 2 | 5 |
| A45 | Vardakas et al. (2015) | 2 | 1 | 1 | 2 | 6 |
| A46 | Lombardi et al. (2015) | 1 | 2 | 0 | 2 | 5 |
| A47 | Alicino et al. (2015) | 0 | 2 | 1 | 2 | 5 |
| A48 | Cubero et al. (2015) | 2 | 0 | 2 | 2 | 6 |
| A49 | Brizendine et al. (2015) | 2 | 1 | 2 | 2 | 7 |
| A50 | Pouch et al. (2015) | 0 | 2 | 1 | 2 | 5 |
| A51 | Japoni-Nejad et al. (2014) | 0 | 1 | 0 | 2 | 3 |
| A52 | Nobari et al. (2014) | 2 | 1 | 0 | 2 | 5 |
| A53 | Simkins et al. (2014) | 2 | 0 | 2 | 2 | 6 |
| A54 | Rastegar-Lari et al. (2013) | 0 | 0 | 0 | 2 | 2 |
| A55 | Kaiser et al. (2013) | 0 | 2 | 0 | 2 | 4 |
| A56 | Giani et al. (2013) | 2 | 2 | 0 | 2 | 6 |
| A57 | Hussein et al. (2013) | 2 | 2 | 1 | 2 | 7 |
| A58 | Mouloudi et al. (2010) | 2 | 0 | 1 | 2 | 5 |
| A59 | Hussein et al. (2009) | 2 | 2 | 1 | 2 | 7 |
| A60 | Gasink et al. (2009) | 2 | 2 | 1 | 2 | 7 |
| A61 | Schwaber et al. (2008) | 2 | 1 | 1 | 2 | 6 |

**Table S5** Global prevalence of carbapenem-resistant Klebsiella pneumoniae infection

|  | **Prevalence (95% CI)** | **No. of study** | **Sample size** |
| --- | --- | --- | --- |
| **Pooled prevalence** | 28.69 (26.53–30.86) | 61 | 513307 |
| **East Asia** | 20.95 (18.99–22.91) | 24 | 343505 |
| China | 19.58 (17.67–21.49) | 23 | 343391 |
| Beijing | 30.73 (15.91–45.55) | 5 | 6889 |
| Hubei | 14.99 (11.09–18.90) | 3 | 1574 |
| Zhejiang | 15.56 (12.33–18.78) | 7 | 286929 |
| Liaoning | 15.22 (5.40–25.04) | 2 | 780 |
| Henan | 48.13 (43.78–52.48) | 1 | 507 |
| Jiangsu | 16.14 (11.87–20.41) | 1 | 285 |
| Shandong | 12.02 (8.05–15.98) | 1 | 258 |
| Hunan | 30.03 (26.65–33.41) | 1 | 706 |
| Shanghai | 31.55 (28.78–34.32) | 1 | 1081 |
| Hebei | 14.26 (13.93–14.58) | 1 | 44382 |
| China Taiwan | 67.54 (58.95–76.14) | 1 | 114 |
| **High-income North America** | 14.29 (6.50–22.08) | 6 | 6879 |
| USA | 14.29 (6.5–22.08) | 6 | 6879 |
| **North Africa and Middle East** | 26.60 (20.26–32.93) | 14 | 3543 |
| Egypt | 57.14 (42.18–72.11) | 1 | 42 |
| Iran | 26.04 (17.14–34.95) | 11 | 1114 |
| Oman | 25.11 (19.47–30.75) | 1 | 227 |
| Tunisia | 15.83 (14.29–17.37) | 1 | 2160 |
| **South Asia** | 66.04 (54.22–77.85) | 4 | 552 |
| India | 67.62 (53.74–81.49) | 3 | 494 |
| Nepal | 60.35 (47.76–72.93) | 1 | 58 |
| **Western Europe** | 42.05 (28.05–50.05) | 13 | 158828 |
| Germany | 0.63 (0.59–0.67) | 1 | 154734 |
| Italy | 40.96 (21.55–60.36) | 6 | 3007 |
| Greece | 70.61 (56.77–84.45) | 2 | 163 |
| Spain | 66.67 (52.41–80.92) | 1 | 42 |
| Israel | 31.98 (18.16–45.8) | 3 | 882 |

**Fig. S1** Tests for publication bias of the 61 publications

**Fig. S2** Sensitivity analysis.


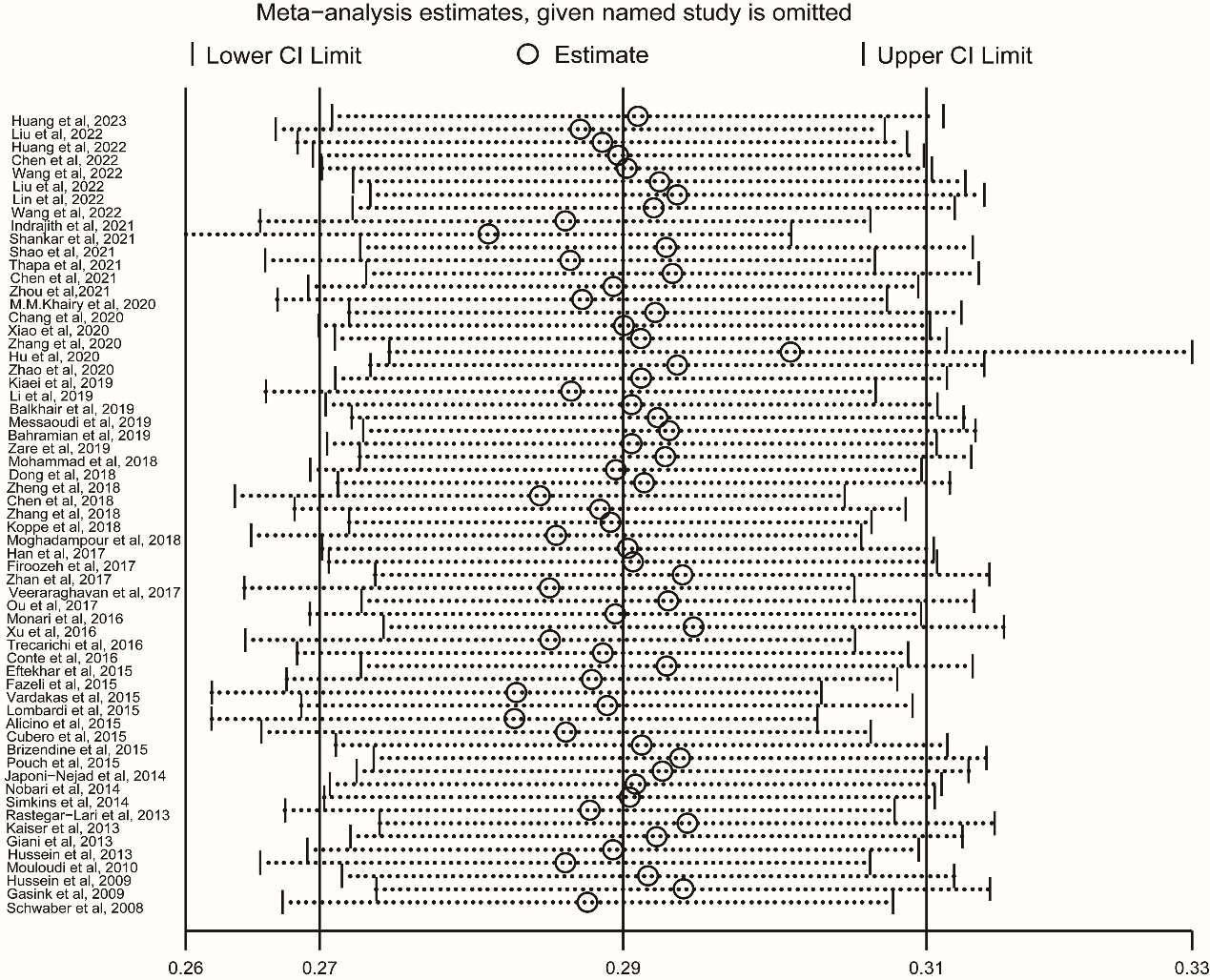

Supplement: ofad649_Supplementary_Data [file ofad649_supplementary_data.docx]
